# Influence of Landmarks on Wayfinding and Brain Connectivity in Immersive Virtual Reality Environment

**DOI:** 10.3389/fpsyg.2017.01220

**Published:** 2017-07-20

**Authors:** Greeshma Sharma, Yash Kaushal, Sushil Chandra, Vijander Singh, Alok P. Mittal, Varun Dutt

**Affiliations:** ^1^Department of Biomedical Engineering, Institute of Nuclear Medicine and Allied Sciences New Delhi, India; ^2^Cluster Innovation Centre New Delhi, India; ^3^Netaji Subhas Institute of Technology New Delhi, India; ^4^All India Council for Technical Education New Delhi, India; ^5^School of Computing and Electrical Engineering and School of Humanities and Social Sciences, Indian Institute of Technology Mandi Mandi, India

**Keywords:** virtual maze, landmarks, wayfinding, left-parietal cortex, event-related desynchronization (ERD), directed transfer function (DTF)

## Abstract

Spatial navigation is influenced by landmarks, which are prominent visual features in the environment. Although previous research has focused on finding advantages of landmarks on wayfinding via experimentation; however, less attention has been given to identifying the key attributes of landmarks that facilitate wayfinding, including the study of neural correlates (involving electroencephalogram, EEG analyses). In this paper, we combine behavioral measures, virtual environment, and EEG signal-processing to provide a holistic investigation about the influence of landmarks on performance during navigation in a maze-like environment. In an experiment, participants were randomly divided into two conditions, Landmark-enriched (LM+; *N* = 17) and Landmark-devoid (LM-; *N* = 18), and asked to navigate from an initial location to a goal location in a maze. In the LM+ condition, there were landmarks placed at certain locations, which participants could use for wayfinding in the maze. However, in the LM- condition, such landmarks were not present. Beyond behavioral analyses of data, analyses were carried out of the EEG data collected using a 64-channel device. Results revealed that participants took less time and committed fewer errors in navigating the maze in the LM+ condition compared to the LM- condition. EEG analyses of the data revealed that the left-hemispheric activation was more prominent in the LM+ condition compared to the LM- condition. The event-related desynchronization/synchronization (ERD/ERS) of the theta frequency band, revealed activation in the left posterior inferior and superior regions in the LM+ condition compared to the LM- condition, suggesting an occurrence of an object-location binding in the LM+ condition along with spatial transformation between representations. Moreover, directed transfer function method, which measures information flow between two regions, showed a higher number of active channels in the LM- condition compared to the LM+ condition, exhibiting additional wiring cost associated with the cognitive demands when no landmark was available. These findings reveal pivotal role of the left-hemispheric region (especially, parietal cortex), which indicates the integration of available sensory cues and current memory requirements to encode contextual information of landmarks. Overall, this research helps to understand the role of brain regions and processes that are utilized when people use landmarks in navigating maze-like environments.

## Introduction

Instantaneous self-updating (i.e., egocentric-updating) is crucial for navigating and wayfinding in an unfamiliar terrain, even with the assistance of sensors or maps (Waller and Hodgson, 2006). During wayfinding, if there are certain prominent features (i.e., landmarks) present on the route, then these features will likely reduce errors and facilitate improved wayfinding (Lee et al., 2006). However, currently, less is known about the behavioral and temporal processes that help quantify the influence of landmarks on wayfinding in novel environments. In this paper, by using experimentation and electroencephalogram (EEG) analyses, we address this problem by investigating certain cognitive and temporal processes that help wayfinding in the presence of landmarks.

Landmarks are visual entities that are perceived as physical objects in space (Epstein and Vass, 2014). These objects are stored in memory as a structure that is based on locations in space and they help in developing route knowledge (Epstein and Vass, 2014). The spatial representation of landmarks is encoded preferentially according to their navigational ability (Wegman and Janzen, 2011). For example, good navigators are significantly more consistent at identifying the most permanent landmarks (Auger et al., 2012). Thus, landmarks influence our ability for successful wayfinding throughout our lives (Jansen-Osmann and Fuchs, 2006; Devlin, 2014). Nevertheless, less attention has been given to the problem of understanding how landmarks influence performance in navigation tasks and what temporal mechanisms are implicated in the presence of landmarks.

Prior research considered landmarks as the building blocks of environmental representation (Siegel and White, 1975; Downs and Stea, 1977). For example, according to the sequential/stage model, Landmark, Route, and Survey (LRS), knowledge of landmarks guide an individual toward environmental patterns which are either perceptually salient or important for environment representation (Siegel and White, 1975). In contrast, cognitive map theory considers spatial relations between landmarks as the basis for navigation, which equally weighs path integration (Gallistel, 1990) with landmarks for maintaining accuracy (O’Keefe and Nadel, 1978; Manns and Eichenbaum, 2009). In all, landmarks are entities that are useful for navigation because they are fixed in space and they can either be distinct objects or extended topographical features such as mountains (Epstein and Vass, 2014).

Electrophysiological and imaging studies have pointed out the importance of landmarks and their corresponding locations in wayfinding (Jansen-Osmann and Wiedenbauer, 2004; Janzen and Jansen, 2010; Wegman et al., 2014). For example, Janzen and Jansen (2010) showed the involvement of Para-Hippocampal Gyrus (PHG) in object and scene recognition. This brain region exhibited higher activity when the objects were encountered at relevant locations as compared to irrelevant ones. On the same note, one EEG study explored the electrophysiological basis of object recognition in a virtual-reality taxi driver game (Weidemann et al., 2009). In this game, participants searched for passengers (neutral stores) and stores (targets or non-targets) during virtual navigation in simulated towns with simultaneous EEG recording. Result showed that theta activity reliably distinguished between the target, non-target, and neutral store views. Frontal-theta oscillatory power was significantly lower for target stores, indicating more-frontal engagement (attention) on the target stores. These findings support the notion of involvement of theta band in object recognition and categorization. However, a deeper investigation is needed that explores the role of different temporal processes that are likely to be associated with navigation in the presence of landmarks.

Furthermore, prior research has made use of immersive virtual environments (i.e., virtual reality) in spatial navigation tasks involving landmarks across different age groups (Jansen-Osmann and Fuchs, 2006; Gazova et al., 2013; Nys et al., 2014). Here, immersion refers to the level of sensory fidelity a virtual-reality (VR) system provides, where the system is both economical and easy to manipulate (Parsons et al., 2013). There has been research involving the use of virtual reality in wayfinding tasks (Bischof and Boulanger, 2003; White et al., 2012), but less attention has been given to EEG analyses in such virtual environments, where people use landmarks for spatial navigation.

In this paper, we overcome this gap in the literature by doing an EEG analyses of wayfinding performance in an immersive virtual environment called Virtual Maze (VM) in the presence and absence of landmarks. In what follows, we first motivate our hypotheses related to behavioral and temporal processes that are likely to be associated with landmarks in navigation tasks. Next, we detail an experiment, where we manipulated the presence or absence of landmarks in a navigation task. We record EEG while participants perform in the navigation tasks. Furthermore, we present the results from our experiment and discuss the implication of our results for behavioral and temporal processes influencing decision-making in wayfinding tasks.

### Hypotheses

In general, landmarks are likely to assist navigators to locate themselves on environmental boundaries (Jansen-Osmann and Wiedenbauer, 2004). Thus, errors would probably reduce when landmarks are present along the route compared to when they are not present (Lee et al., 2006). We believe that, in the presence of landmarks, wayfinding performance would improve. Thus, our first hypothesis is:

H1: Wayfinding in a virtual environment would be less error-prone when landmarks are present compared to when landmarks are absent.

Considering brain activity, the literature shows that whenever object processing is involved, activity in the left hemisphere, especially in the parietal region, is quite evident (van Asselen et al., 2009). van Asselen et al. (2009) demonstrated that the activation in the left-hemispheric parietal cortex had been prominent when both categorical and coordinate information led to object-location binding in memory. In literature, two kinds of spatial relation/representation have been discriminated for object-location binding in memory: coordinate spatial relation and categorical spatial relation. The coordinate spatial relation is a precise metric representation while categorical spatial relation is a general representation. Also, egocentric and allocentric spatial representations are also important for understanding object-location binding in memory. Egocentric representation is encoding of spatial information about a person’s location and orientation; whereas, allocentric representation is encoding of spatial information about other objects, independent of the location or orientation of the observer (Klatzky, 1998). Subsequently, there are four possible combinations of spatial memory representations: (a) egocentric–categorical (the landmark is in left of you); (b) egocentric–coordinate (the landmark is 0.5 m from you); (c) allocentric–categorical (the landmark is to the right of the wall); and, (d) allocentric–coordinate (the landmark is 0.5 m from the wall) (Baumann et al., 2012). Baumann et al. (2012) reported a significant stronger activity in the left-lateral and medial-parietal cortex as well as in the left-middle temporal gyrus when participants were engaged in the categorical object-location binding in memory. Given the implication of brain’s left-hemisphere in the categorical object-location binding, our second hypothesis is:

H2: Left-hemispheric brain regions would be implicated (a significant left hemispheric activation) when landmarks are present compared to when they are not present.

When functional connectivity between a pair of channels are considered then it is important to understand the associated wiring cost for an efficient network topology (the layout pattern of interconnections). Wiring cost is defined as the fixed cost of making anatomical connections between neurons, often approximated by the wiring volume of anatomical connections such as axonal connection. An efficient network is often used as a measure of the overall capacity for parallel information transfer and integrated processing between connections (Bullmore and Sporns, 2012). These facts suggest that it would be easy for a participant to perform wayfinding task when landmarks are available along the route compared to when they are not available. When highest degree of connections are established between a channel with other channels (where the channel is considered as a hub), our third hypothesis is:

H3: Lower number of hubs would be active when landmarks are present compared to when they are not present.

In the next section, we report an experiment involving a wayfinding task with and without landmarks in order to test our hypotheses. Overall, as per our hypotheses, we believe that landmarks would facilitate wayfinding and that there would be stronger left-hemispheric activations and lower number of hubs activated in the presence of landmarks.

### Experiment: Influence of Landmarks on Wayfinding in a Virtual Maze Environment

In this section, we report an experiment with human participants, where people were asked to navigate a virtual-maze environment in the presence or absence of landmarks. We performed both behavioral and electrophysiological analyses of data collected in order to test our hypotheses.

## Materials and Methods

### Participants

Thirty-five healthy human adults (30 males) with normal or corrected-to-normal vision gave informed written consent before participating in the experiment. All participants were right-handed and possessed no antipsychotic medication according to self-reports. Mean age was 24.75 years (age ranged from 21 to 30 years). All participants were engineering students and data was recorded at the Institute of Nuclear Medicine and Allied Sciences, New Delhi. Participants were compensated with a travel allowance and a participation certificate at the end of the study.

### Virtual-Maze Task

**FIGURE 1:**
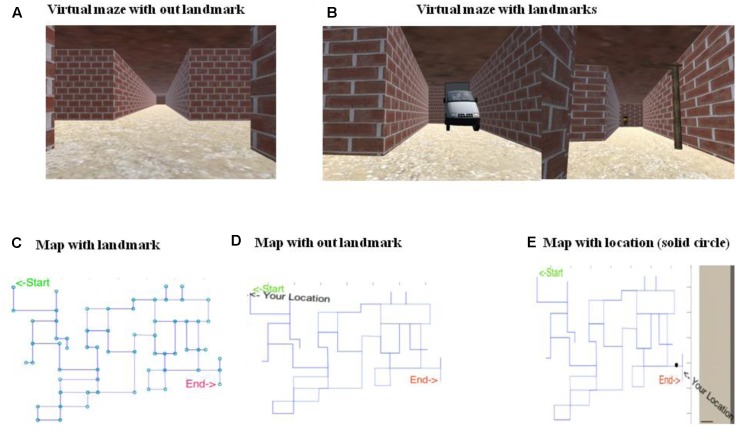
Snapshots of virtual Maze (VM) along with the maps. **(A)** Shows the VM without landmarks. **(B)** Shows the VM with landmarks. **(C)** Shows the map with landmarks, however, this version of map was never used in the experiment. Here, circle represent the position of landmarks which are only present at junction. The original map is shown without circle. **(D)** Shows the map without landmarks, which was shown to participants. **(E)** Shows position of the navigator on the map represented by solid black circle which was accessible to the participants.

The primary objective of the Virtual-Maze task was to go from an initial point to a goal point by navigating a maze using the shortest possible route. The navigation was performed in a virtual environment, where the environment was built using Unity 5.0 software. A route in the Virtual-Maze (VM) environment was shown as a brick corridor with a plain ceiling, where there was sufficient contrast between the floor, ceiling, and walls (see **Figures 1A,B**). There were multiple light sources used in the task to avoid directional cues from shadows. A map of the maze was provided to participants before they started navigating in the VM task (see **Figures 1C,D**). This map may assist participants to plan their route in the VM task. Participants navigated through the maze using a wireless Joystick. Camera in the VM was positioned 1.75 m above the floor corresponding to an average height of the human. Movement speed was fixed to 8 km/h to match regular walking speed. The VM was displayed via an Oculus Rift Development Kit 2 (DK2) through Mac-Book Air, 13-inch screen, 1.4 GHz Core i5 processor with a resolution of 1920 pixels × 1200 pixels. The Oculus DK2 combines a magnetometer, an accelerometer, and a gyroscope to track head movement across all three dimensions accurately. The Oculus emulated real-world movement in virtual reality, i.e., the visual scene changed with the orientation of the head. The Head Mounted Display (HMD) provided a 100-degree horizontal field of view with a 75 Hz refresh rate. The Oculus Rift presented two images, one for each eye, generated by two virtual cameras separated by a short distance. The separation of the lenses was adjustable by a dial on the bottom of the device to accommodate a broad range of interpupillary distances.

In the VM task, certain common objects (like electric post or car) were used as landmarks (all objects were familiar to participants). Each junction in the virtual-maze task had either one or multiple (maximum three) choices to choose between for navigation. There were three routes to reach the end-point, where one of the three was the optimal (shortest) path to navigate the maze. Thus, one could calculate the deviation from the optimal path for a participant at each junction point. The deviation from the optimal path led participants taking more time and feeling more disoriented. Path-lengths between junctions varied, some were equal while others were longer. The goal-point was marked with an object shape resembling a green colored dog shape. Participants had to touch this shape to mark their reaching the goal point in the VM task.

### Behavioral Measures

Five participants were uncomfortable with the immersive VM; therefore, only 30 participants further took part in the experiment. The Unity software automatically measured participant’s position in *x*–*y* coordinates and heading orientation (yaw). These data were automatically output into a text file and converted by custom software that plotted the navigation path onto a 2D map of the space. The software then calculated performance score. Performance score included coordinates of the path traveled by the participants, proportions of repetitions (*R*_P_), total time taken for navigation (*T*_T_), average number of map views (*M*_A_), average time spent to view a map (*M*_T_), Number of turns (*N*_T_), and total distance covered (*T*_D_). *R*_P_ was defined as the ratio of a total number of repetitions of the path and the sum of repetitions and non-repetitions of the path. *M*_T_ was an average time spent by the participants to view the map. M_A_ was defined an average number of times map was viewed during wayfinding from the start-point to the end-point. *T*_T_ was the time taken from the start-point to the end-point. Maximum allotted *T*_T_ was 15 min; but, if participants failed to reach the end-point within this time, then the VM automatically disappeared. *N*_T_ was defined as the total number of turns taken by participants during wayfinding.

### EEG Signal Acquisition

The EEG data were recorded through Ag/AgCl electrodes from 64-electrode points according to the extended 10–20 electrode placement system against the mastoid reference. An eego^TM^sports EEG acquisition system (ANT Neuro, Enschede, Netherlands) was used. The EEG signals were sampled at 1024 Hz. The impedance was kept below 5 kΩ. To minimize artifacts, wave guard cap was used which was actively shielded. Active shielding technology protected the central components of EEG cables against artifacts generated by body and cable movements (Cheron et al., 2016). The EEG signals were recorded during resting state and navigation. EEG data were visually inspected for eye blink and muscular artifacts in ‘asa^TM^ pro’ software. Artifact rejection was performed using independent component analysis (ICA, Onton and Makeig, 2006) from EEGLAB (Delorme and Makeig, 2004). A notch filter was used to remove 50-Hz power line interference and a Butterworth band-pass filter (between 4 and 8 Hz) was used to calculate theta frequency band.

The percentage change in theta power between the baseline condition (2-min eye open) and during the navigation (time-locked for landmarks in ‘LM+’) was computed. Out of the total 2 min taken, a reference interval (R) comprised the time from the 30th second to the 90th second. The activation interval (A) consisted of 1 min period during navigation. The formula used for calculating %ERD (Event related desynchronization) was the following (Pfurtscheller and Da Silva, 1999):

(1) $$\%ERD=\frac{R-A}{R}*100$$

The negative values (ERD) represent cortical activation (desynchronization), whereas positive values [Event-related synchronization (ERS)] portray cortical deactivation (synchronization). Theta ERD/ERS were computed because of their relevance in spatial navigation (White et al., 2012) and in spatial memory processing (Cornwell et al., 2008).

For better interpretation of percentage ERD, electrode channels were grouped into eight topographical regions of interest (ROI): Left Anterior Inferior (LAI) : F7 AF7 AF3 F5 FC5 C5 FT7; Left Anterior Superior (LAS): F3 F1 C1 C3 FC1 FC3; Left Posterior Inferior (LPI):TP7 P7 PO7; Left Posterior Superior (LPS): CP3 CP1 P1 P3 P5 PO3; Right Anterior Inferior (RAI): AF4 AF8 F6 F8 FC6 FT8 C6; Right Anterior Superior (RAS): FCZ F2 F4 C4 FC2 C2 FC4; Right Posterior Inferior (RPI): PO8 P8 TP8; Right Posterior Superior (RPS): CP2 CP4 P6 PO4 P4 P2 (Weidemann et al., 2009).

### Directed Transfer Function (DTF)

Directed transfer function (DTF) is used to estimate directed connectivity and does not produce spurious connections. For biomedical time series where the contribution of noise is quite high, the estimates of connectivity based on multichannel autoregressive model (MVAR), especially DTF, are recommended. DTF is designed to find the relationship between two channels about all the other channels of a system being analyzed. The function is based on the properties of the transfer function of the whole multivariate structure of a process of *k* channels, the MVAR (Granger, 1969, 1981) and normalized version of DTF is defined as:

(2) $${DTF}_{j\to i}^{2}(f)=\frac{{\left|H_{ij}(f)\right|}^{2}}{\Sigma_{m=1}^{k}\,|H_{ij}{\left(f\right)}^{2}|}$$

Where H_ij_(f) is called the transfer matrix of the system between channel *i* and *j*. It contains information about all relations between data channels in the given set including the phase relations between signals. The DTF describes the causal influence of channel *j* on channel *i* at frequency *f* (descriptor of flow of information). It takes values from 0 to 1, producing a ratio between the inflow from channel *j* to channel *i* to all the inflows to channel *i* (Blinowska, 2011).

### Experimental Design and Dependent Measures

Participants were randomly assigned to one of the two between-subject conditions: LM+ or LM-. Participants were matched on age and education. Seventeen participants were assigned in the LM+ while 18 participants were kept in the LM-. In the LM+ condition, the participants navigated around the maze when landmarks were present along the route. In the LM- condition, the participants navigated around the maze when the landmarks were absent along the route. Our main intention was to evaluate the influence of one independent measure (the landmark) in this experiment. To address hypothesis 1, behavioral measures were compared when landmarks were present and absent. Furthermore, each condition was compared with the optimal path and turns in VM. To address hypothesis 2, we compared brain activity (each ROI) with the LM+ and LM- to identify prominently activated brain regions when landmarks were present. In the LM+, all the familiar objects were used as landmarks. Total fifty-three landmarks were used in the LM+ (20 landmarks on the optimal route). While in the LM- condition, no landmark was used. To address hypothesis 3, we compared DTF graph for the significant ROI to understand brain connectivity between a pair of channels as well as to identify the relatively active channel in a particular ROI between two conditions.

### Procedure

**FIGURE 2:**
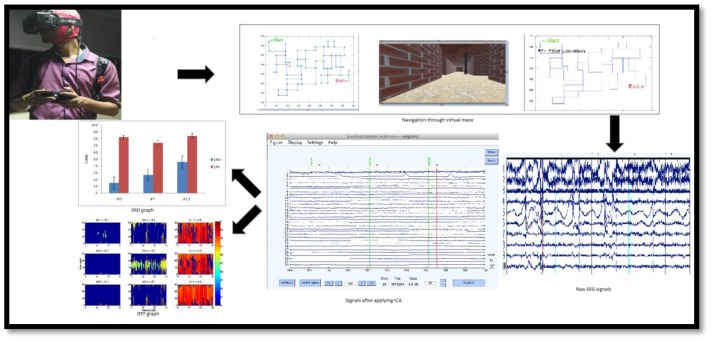
Experimental procedure from data acquisition to data analysis. **(Top)** Navigation in VM (right), displaying map for planning then navigation with activated GPS in the VM for spatial updating by subject (left) wearing EEG headset and oculus rift (HMD) with joystick for movement. **(Bottom)** Data Analysis from data segmentation (right, separating Baseline from task), artifact-removal (middle, Applying ICA), and feature extraction for particular ROI (left, graph for %ERD and DTF).

The experiment was conducted in a noise-free, dim-lit, and closed room environment. Before performing in a condition, participants were made habituated with the computer controls for movement and the virtual-maze task. Initially, the participants had to plan their route from the starting-point to the end-point in the virtual-maze task using a map (participants could peruse the map for as long as they wanted to before the start of their performance in the virtual-maze environment). It should be noted that both conditions received similar maps, i.e., in both conditions participants did not see landmarks on the map. Subsequently, after planning the route, all the participants were asked to navigate through the virtual-maze. Participants could check the map at any point while traveling in the virtual-maze by pressing a corresponding key on the joystick. The flow of experiment is shown in **Figure 2**. In the flow, the whole procedure is mentioned from data acquisition to the results of the study, including employed VM and the analysis method.

The map showed a point indicating the location of the participant on the maze (**Figure 1E**). Participants were given 15-min to complete the maze task after planning their route from the starting point to the end-point. When participants reached end-point, the maze disappeared, indicating the end of the task.

## Results

### Behavioral Outcome

**Table 1** shows the descriptive statistics for the performance scores in the LM+ and LM- conditions. Independent sample *t*-tests were applied to the behavioral measures to check significant differences between conditions. *R*_P_ was found significantly lower in the LM+ condition compared to the LM- condition [*t*(28) = 2.92, *p* = 0.023, *r* = 0.39]. Similarly, *M*_T_ was found to be significantly lesser in the LM+ condition compared to the LM- condition [*t*(28) = 2.24, *p* = 0.048, *r* = -0.18]. Overall, these results show that participants had a lower number of repetitions and lesser average time spent during a map view in the LM+ condition compared to the LM- condition. Thus, as per our expectation in H1, participants could successfully integrate landmark information in their decision-making in the LM+ condition compared to the LM- condition.

**Table 1:** Mean (*SD*) for performance variables in Virtual Maze (VM).

Performance Variables	Conditions in Virtual Maze	
	
	With Landmarks (LM+) Mean (*SD*)	Without Landmarks (LM-) Mean (*SD*)
*R_p_^∗^*	0.32 (0.16)	0.51 (0.16)
*T*_T_ (sec.)	316.18 (232.37)	474.91 (226.64)
*M*_A_	23.00 (23.34)	38.50 (20.21)
*M*_T_^∗^ (sec.)	64.18 (38.69)	123.16 (52.03)
*N*_T_	18.88 (3.40)	19.45 (4.61)
*T*_D_ (meter)	1202 (331.07)	1332 (461.85)


*Behavioral variables during navigation were measured for each participant and averaged group means. *R*_*P*_, proportions of repetitions; *T*_*T*_, total time taken for navigation; *M*_*A*_, average number of map views; *M*_*T*_, average time spent to view a map; *N*_*T*_, Number of turns; *T*_*D*_, Total distance covered. ^∗^*p* < 0.05.*

### Cortical Activation (%ERD)

**FIGURE 3:**
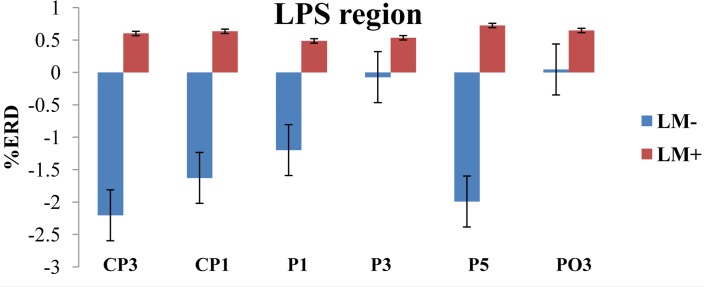
%ERD graph for LPS region. The negative axis shows synchronization while the positive axis shows desynchronization. For LM+, more desynchronization was observed for LPS region.

**FIGURE 4:**
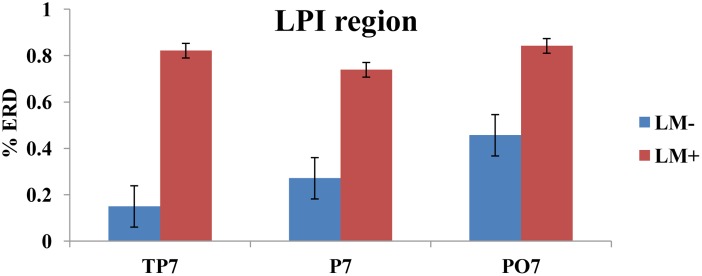
%ERD graph for LPI region. Bar plot shows desynchronization for both LM+ and LM–. For LM+, more desynchronization was observed in LPI region.

For individual ROIs, univariate ANOVA was applied by considering condition (LM+, LM-) as an independent variable and each ROI as dependent variables. Results showed that two out of the eight ROIs (LPI and LPS) had significant differences between the LM+ and LM- conditions. In the LPS region, the %ERD was higher in the LM+ condition compared to the LM- condition [LM+: *M* = 0.60, *SE* = 0.66 > LM-: *M* = -1.17, *SE* = 0.54; *F*(1,150) = 4.261, *p* = 0.041, η^2^ = 0.035]. In the LPI region, the %ERD was higher in the LM+ condition compared to the LM- condition [LM+: *M* = 0.80, *SE* = 0.10 > LM-: *M* = 0.29, *SE* = 0.088; *F*(1,75) = 13.34, *p* = 0.001, η^2^ = 0.187]. Independent *t*-tests were applied to identify significant channels in a particular ROI. As shown in **Figure 3**, for the LPS region at PO3, the %ERD was higher in the LM+ condition compared to the LM- condition [*t*(28) = 1.96, *p* = 0.040, *r* = 0.24]. Also, as shown in **Figure 4**, for the LPI region at TP7 and PO7, the %ERD was higher in the LM+ condition compared to the LM- condition [*t*(28) = 2.86, *p* = 0.013, *r* = 0.06 and *t*(28) = 3.22, *p* = 0.007, *r* = 0.54, respectively]. Results were not significant for the channels CP3, CP1, P1, P3, and P5 in the LPS region; and, for the channel P7 in the LPI region.

Participants in the LM+ condition showed higher %ERD in theta band during wayfinding compared to the participants in the LM- condition. Higher %ERD could be interpreted as activation of the respective cortical area. Prior studies had showed an increase in the theta band power when landmarks were visible during spatial navigation task in a VM, which reflected encoding of the landmark location (Kober and Neuper, 2011). Therefore, for LPS and LPI regions, higher brain activity was observed when landmarks were available on the route. This result demonstrated that left-hemispheric brain especially parietal lobe and its associates were found to be more connected with the landmark processing. Thus, as per our expectation in H2, left-hemispheric brain regions would be implicated (i.e., a significant left-hemispheric activation) when landmarks are present compared to when they are not.

**FIGURE 5:**
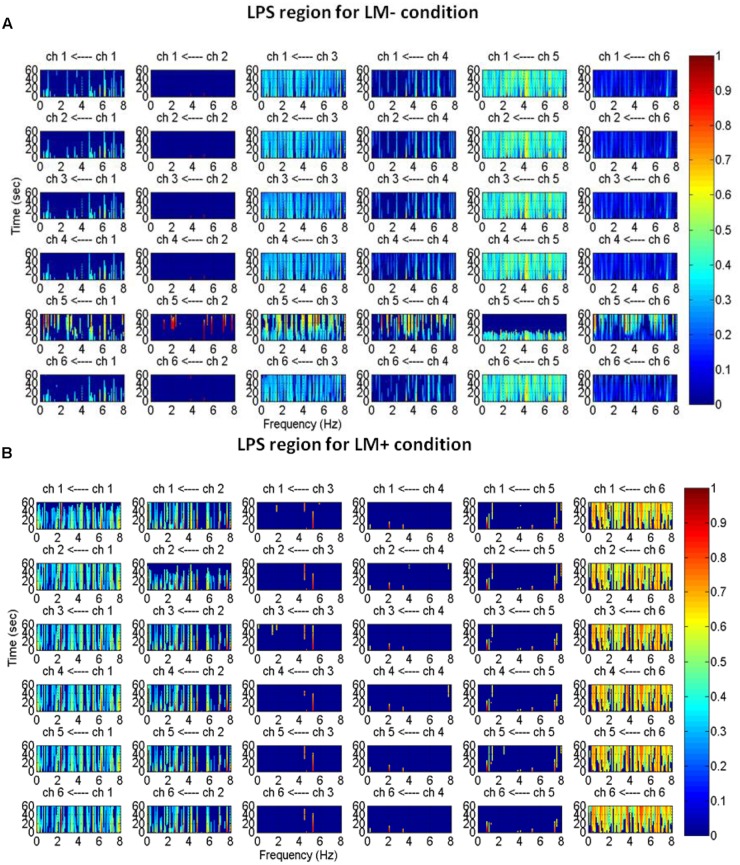
Estimated average normalized DTF of data recorded from six electrodes (LPS region) during Navigation. Here, ch1, ch2, ch3, ch4, ch5, and ch6 represents CP3, CP1, P1, P3, P5, PO3, respectively. **(A)** DTF graph for the LM– condition. **(B)** DTF graph for the LM+ condition.

**FIGURE 6:**
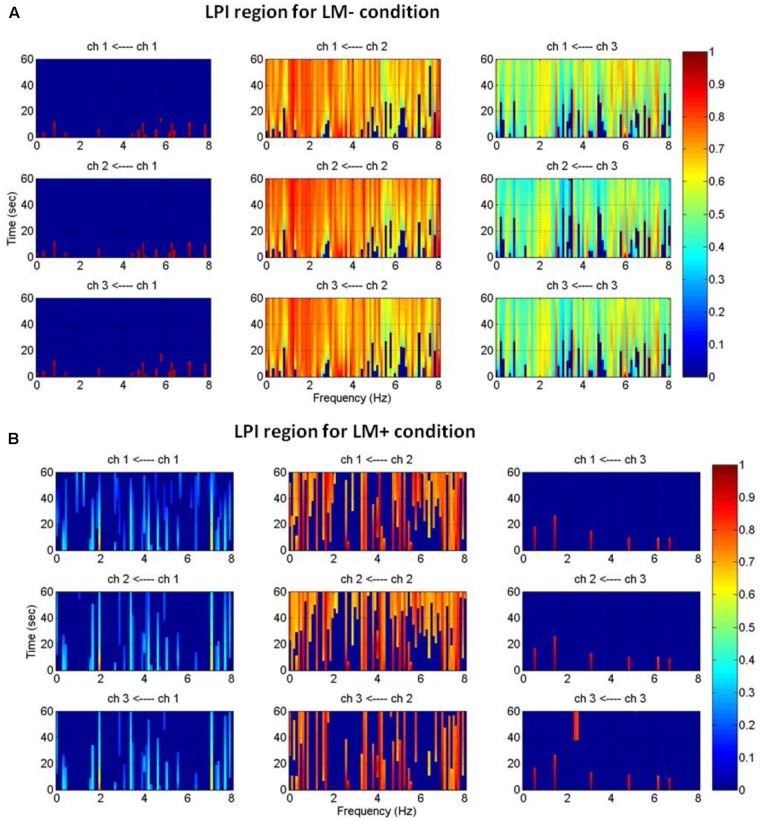
Estimated average normalized DTF of data recorded from three electrodes (LPI region) during Navigation. **(A)** Shows DTF graph for the LM– condition. **(B)** Shows DTF graph for the LM+ condition. Here, ch1, ch2, and ch3 represents TP7, P7, and PO7, respectively.

An active channel was considered as a hub when it established the highest degree of connections with other channels in LPS and LPI regions. Hubs were computed by applying DTF, which informed about the direction of flow between two channels. DTF graph was plotted by considering the time of the event on the *y*-axis (between 0 and 60 s) with the frequency of activation on the *x*-axis (between 0 and 8 Hz). The direction of flow of information between two channels was shown by the head of the arrow. For example, ch6→ch1 showed the flow of information from ch6 to ch1. The strength of information flow between two channels was pictorially represented by a continuous color scale on the right-side to the DTF graphs. In the scale, the red color represented higher activations while blue color represented no activations. In the LPS region, LM- exhibited higher activity at P5 and P1 (**Figure 5A**); while LM+ exhibited higher flow activity from PO3 channel to other channels (**Figure 5B**). Also, in the LPI region, LM- showed higher activity at P7 and PO7 channels (**Figure 6A**), whereas LM+ showed higher activity at P7 channel (**Figure 6B**). The lower number of active hubs in the LM+ condition compared to LM- condition suggested reduced activity under the influence of landmarks. In other words, participants required active hubs to maintain egocentric and allocentric categorical updating when landmarks were absent. Thus, as per our expectation in H3, the lower number of hubs were active when landmarks were present compared to when they were not.

## Discussion

The aim of the present study was to explore an effect of landmarks on the performance as well as on the brain regions during navigation in a completely immersive virtual environment (VM). The effect was elucidated by addressing three specific hypotheses: (i) Landmark facilitates successful wayfinding, (ii) There would be stronger left-hemispheric activation in the presence of landmarks, and (iii) There would be a fewer active channels when landmarks were present compared to when they were absent. Behavioral measures showed that participants committed few errors and took shorter time when landmarks were available compared to when landmarks were not available. Thus, landmarks facilitated successful wayfinding. The EEG result revealed that participants had higher left-hemispheric activations, especially in the parietal region when landmarks were present. Also, there were fewer hubs in the LM+ condition compared to LM- condition, which indicated that participants deployed fewer cognitive resources to integrate landmark knowledge onto the route knowledge. Thus, landmarks processing showed stronger influence on the brain regions as assessed by higher %ERD and fewer number of hubs in the LM+ condition compared to the LM- condition.

Behavioral results showed that participants performed better by committing fewer errors and taking shorter time to complete the task in the LM+ condition compared to LM- condition. This result confirmed the first hypothesis that landmark would facilitate successful wayfinding, and; this finding aligns with previous studies (Jansen-Osmann and Wiedenbauer, 2004; Jansen-Osmann and Fuchs, 2006; Wegman et al., 2014). An earlier rodent study (Youngstrom and Strowbridge, 2012) confirmed the pivotal role of landmarks in the spatial representation for successful wayfinding. The study demonstrated that visual cues such as landmarks alone could be employed to form spatial representation during wayfinding; henceforth, landmarks facilitate successful wayfinding in the rodents. Similarly, in our case, landmarks facilitate the participant to tag the route which in turn reduce the chances of disorientation. When participants were in the LM+ condition, they had lower disorientation. This disorientation might occur because landmarks on the route assisted in the formation of spatial representation. Spatial representation using landmarks assist participants to determine their current position and heading to infer the correct path. If participants had chosen the wrong turn then they could avert moving in the wrong direction by viewing the landmark en-route and the position (oneself) on the map. Since the map was similar in both the conditions, the cost incurred by disorientation (longer distance) was saved by landmarks in the LM+ condition. Altogether, behavioral measures supported the first hypothesis.

There is a growing literature examining theta wave in the human brain using spatial navigation task in virtual reality using EEG, magnetoencephalography (MEG), and functional magnetic resonance imaging (f-MRI) (Kahana et al., 1999; Bischof and Boulanger, 2003; Janzen and Van Turennout, 2004; Weidemann et al., 2009; Wegman and Janzen, 2011). EEG results showed task dependent theta activity, which was found related to encoding and retrieval of spatial information in the VM (Bischof and Boulanger, 2003). In a study, theta wave was identified as an important variable in object recognition and categorization (Weidemann et al., 2009). In the same manner, the present study demonstrated significant theta activity through %ERD when landmarks were available, confirming the results of previous studies. Despite the large differences in the methods, theta activity was present during wayfinding task in the VM and it increased when landmarks were present.

Additionally, this study indicated that higher %ERD in the left hemisphere plays a role in the landmark processing. Previous studies reported hemispheric lateralization in spatial cognition such as establishing spatial relations between objects (van Asselen et al., 2009; Amorapanth et al., 2010). For instance, Amorapanth et al. (2010) demonstrated that neural processing of categorical spatial relations between objects was distinct from the processing of the identity of objects. They found greater activity in superior and inferior parietal cortices (especially on the left) when attending to the categorical spatial relations compared with attending to the identity of objects. This observations implied that left-hemisphere is biased toward processing categorical spatial relations and the right-hemisphere is biased toward processing coordinate spatial relations. Importantly, left-hemisphere [especially posterior parietal cortex (PPC)] mediated both categorical and coordinate spatial relations. Another lesion study exhibited significance of left parietal cortex for binding object-location (categorical and coordinate) information in memory (van Asselen et al., 2009). Similarly, in the present study, we found activation of the left parietal region (LPI and LPS) when landmarks were available on the route. A higher activity in the left parietal region showed that the participant in the LM+ condition was able to recognize the landmark (object recognition) in route, thereby indicating binding of landmarks and their positions (either or both categorical and coordinate) in memory for the LM+ condition compared to the LM- condition. Extending role of PPC suggested its involvement in the translation of the participant’s position from a world-centerd coordinate system (allocentric information) generated by hippocampus into the body-based coordinates of locomotors actions (egocentric information) (Whitlock et al., 2008). This step seems necessary for planning the next movement in a navigational sequence. In the same manner, participants in the LM+ condition used left parietal cortex and its associates to translate categorical and coordination information of landmarks into egocentric information to decide movements at the junction.

Similarly, the role of the left medial parietal lobe (MPL) has been recognized in active spatial navigation. One imaging study emphasized that left MPL was crucial for maintaining the representation of heading direction which is essential in constructing and recalling links between landmarks and directional information (Baumann and Mattingley, 2010). In this study, authors used navigation task in the VM, consisting of two phases. In the first phase, learning phase, participants were familiarized with the maze layout and presented pairs of landmarks depicting either the same heading direction (e.g., north–north) or different heading directions (e.g., west–north). Participants were told to navigate as quickly and directly as possible to the location of one of the landmarks. In the second phase, test phase, participants were shown static images of landmarks and told to press the button if displayed image was on the left or right of the center point of the maze. The result showed reduced activity in the MPL when presented images were in the same heading as they were learned in the learning phase for a VM. Thus, the study implicated that key landmarks were encoded in a manner that reflected the allocentric directions in which they were observed during learning. Our findings supported these prior observations by demonstrating that, in human PPC and MPL, landmarks were encoded on perceived heading (by GPS on map) in memory. Such encoding conceivably facilitated the wayfinding as it integrated full action sequence associated with route traversal with the geometric appearance of the track (in LM- and LM+ conditions) and landmarks of the track (in LM+ condition only). Summing up, greater theta activity was observed during wayfinding in the VM, reflecting the importance of %ERD in the study. Also, Left parietal regions (PPC and MPL) showed greater activity in the landmark processing, exhibiting significance of LPI and LPS regions. Altogether, %ERD results supported the second hypothesis.

Considering functional connectivity, a higher number of the active electrodes in LPI and LPS regions were found in the LM- condition compared to the LM+ condition using DTF method. A higher number of active electrodes represented the extra connection to a network, each added connection represented an incremental cost regarding wiring volume and operational resources (Bullmore and Sporns, 2012; Papo et al., 2014). Participants in the LM- condition had higher chances of disorientation in the VM which increased the re-wiring cost associated with such frequent cognitive demands. This, in turn, suggested efficient network topology (utilizing fewer cognitive resources) for the participants in the LM+ condition compared to the LM- condition, which was in-line with the behavioral outcome where participants performed better in the LM+ condition compared to the LM- condition. Thereby, this observation provided underpinning for effortlessly integrated information in the LM+ condition, suggesting a fewer number of activated channels. Altogether, results from DTF supported the third hypothesis.

## Conclusion

Consistent with the previous results, landmarks improved wayfinding. Computational and signal processing methods exhibited activity in LPI and LPS regions, which integrated categorical and coordinate representation together and maintained perceived heading direction that is essential for successful wayfinding. Left parietal region or left parietal junction played a critical role in the binding of object-location memory for egocentric and allocentric spatial relations. Also, it showed involvement in the spatial transformation which was important to translate egocentric information to allocentric information and vice versa. DTF methods showed active electrodes in a particular ROI. A fewer number of hubs in the LM+ condition showed brain networks efficiency during wayfinding when landmarks were available on the route. More broadly, our findings indicated that left parietal cortex (posterior and medial) plays a key role in binding categorical and coordinate spatial information in memory, maintaining heading direction, and translating allocentric information to egocentric and vice versa. The current study suggests the importance of the signal processing techniques and completely immersive virtual environments to measure cortical dimensions for landmarks in a navigation environment. Thus, it sheds new light on why the presence of landmarks facilitate wayfinding. Together, the study supports various prior imaging studies where the role of PPC and MPL had been identified in the spatial navigation.

Future work will need to examine the effect of landmarks on wayfinding when other sensory cues such as auditory and tactile are available to the observer. The cellular mechanisms within LPI and LPS regions which support the object recognition and transformation in a VM also need to be investigated. It will also be interesting for future studies to explore how the left hemisphere provide support for integrating landmark knowledge on the route knowledge; and, to investigate whether the presence of landmarks permits an individual to switch between strategy, reflecting individual differences.

## Ethics Statement

This study was carried out in accordance with the recommendations of ‘Ethical guidelines, Ethical committee, INMAS’ with written informed consent from all subjects. All subjects gave written informed consent in accordance with the Declaration of Helsinki. The protocol was approved by the ‘Ethical committee, INMAS.’

## Author Contributions

GS: She is the key contributor to the paper. She has designed the study, collected data, analyzed, and write the paper. YK: He is the key person in the development of the virtual environment. SC: He conceptualizes the idea of navigation in an immersive virtual environment. He manages the participants of the study. VS: He helps in the analyzing of the data and its interpretation. AM: He helps in the conduction of the study, analyzing the data and paper writing. VD: He contributes to the restructuring of the manuscript with the grammar editing.

## Conflict of Interest Statement

The authors declare that the research was conducted in the absence of any commercial or financial relationships that could be construed as a potential conflict of interest.
