# Corrigendum: Influence of Landmarks on Wayfinding and Brain Connectivity in Immersive Virtual Reality Environment

**DOI:** 10.3389/fpsyg.2017.01514

**Published:** 2017-09-04

**Authors:** Greeshma Sharma, Yash Kaushal, Sushil Chandra, Vijander Singh, Alok P. Mittal, Varun Dutt

**Affiliations:** ^1^Department of Biomedical Engineering, Institute of Nuclear Medicine and Allied Sciences New Delhi, India; ^2^Cluster Innovation Centre New Delhi, India; ^3^Netaji Subhas Institute of Technology New Delhi, India; ^4^All India Council for Technical Education New Delhi, India; ^5^School of Computing and Electrical Engineering and School of Humanities and Social Sciences, Indian Institute of Technology Mandi Mandi, India

**Keywords:** virtual maze, landmarks, wayfinding, left-parietal cortex, event-related desynchronization (ERD), directed transfer function (DTF)

In the original article, we neglected to add information regarding one of co-author of this paper related to the project funding. Please add following sentence in the funding section of manuscript. The sentence is “Dr. Varun Dutt is a principal investigator on one of the sub-projects under DRDO project ST-14/DIPR-734.” The authors apologize for this oversight. This error does not change the scientific conclusions of the article in any way.

## Conflict of interest statement

The authors declare that the research was conducted in the absence of any commercial or financial relationships that could be construed as a potential conflict of interest.

